# Deformation Activity Analysis of a Ground Fissure Based on Instantaneous Total Energy

**DOI:** 10.3390/s19112607

**Published:** 2019-06-08

**Authors:** Xianglei Liu, Shan Su, Jing Ma, Wanxin Yang

**Affiliations:** 1 Key Laboratory for Urban Geomatics of National Administration of Surveying, Mapping and Geoinformation, Engineering Research Center of Representative Building and Architectural Heritage Database, the Ministry of Education, Beijing University of Civil Engineering and Architecture, Beijing 100044, China; liuxianglei@bucea.edu.cn (X.L.); sushan787426@163.com (S.S.); 2 Beijing Institute of Geo-enginnering and Exploration, Beijing 100048, China; 3 Department of Geography, University College London, Gower Street, London WC1E 6BT, UK; w.yang.17@ucl.ac.uk

**Keywords:** activity analysis, ground fissures, shape acceleration array (SAA), extreme-point symmetric mode decomposition (ESMD), instantaneous total energy

## Abstract

This study proposes a novel instantaneous total energy method to perform an activity analysis of ground fissures deformation, which is calculated by integrating the extreme-point symmetric mode decomposition (ESMD) method and kinetic energy based on the time-series displacement acquired by shape acceleration array (SAA) sensors. The proposed method is tested on the Xiwang Road fissure in Beijing, China. First, to fully monitor the hanging wall and footwall of the monitored ground fissure, a 4 m-long SAA in the vertical direction and an 8 m-long SAA in the horizontal direction were embedded in a ground fissure to obtain an accurate time-series displacement with an accuracy of ±1.5 mm/32 m and a displacement acquisition frequency of once an hour. Second, to improve the accuracy of the activity analysis, the ESMD method and Spearman’s rho are applied to perform signal denoising of the original time-series displacement obtained by the SAA sensors. Finally, the instantaneous total energy is obtained to analyze the activity of the monitored ground fissure. The results demonstrate that the proposed method is more reliable to reflect the activity of a monitored ground fissure compared to the time-series displacement.

## 1. Introduction

Ground fissures are a type of ground deformation that are dangerous because they can be concealed and cause direct damage to many types of engineering construction projects [1,2,3], such as linear engineering projects, water conservation facilities, and urban buildings. In addition, they can seriously restrict urban planning, the effective land use, the exploitation of underground water, and the development of underground space in cities [4]. Therefore, it is urgent to develop methods to analyze the activity and deformation mechanism of ground fissures to ensure the safety of public and private infrastructure projects.

A significant amount of human and financial resources has been invested to investigate different technical measures to more thoroughly understand the activity and deformation mechanism of ground fissures [5,6,7]. The geodetic technique is a commonly used surveying approach that uses leveling, the global positioning system (GPS), and interferometry synthetic aperture radar (InSAR) technologies to detect and monitor ground fissures [5,8]. Leveling is a traditional, ground deformation monitoring technique that can provide high accuracy surface subsidence measurements at selected locations, which is simple to operate and uses inexpensive equipment [9]. The highly accurate digital elevation model (DEM) can also be used to reflect the overall settlement of a monitored area [6]. However, DEM, produced by leveling, requires an extensive artificial field survey that takes a long period of time, and it is also difficult to obtain complete ground fissure deformation information from time and space [7]. The GPS technique has the advantages of high accuracy and simultaneous three-dimensional (3D) positioning to obtain an absolute 3D deformation map of ground points in a region [10]. However, the density of GPS monitoring points deployed is limited due to various factors [5,11]. Due to the advantages of high spatial resolution and all-day and all-weather observation, InSAR technology has been successfully applied to many geological hazards monitoring processes in recent decades [5,12,13,14,15,16]. The InSAR technique can provide deformation sequence results of a monitored ground fissure in the form of an average annual deformation rate map that allows for the detection of ground fissure activity [5]. However, due to a lack of accurate absolute position and deformation references coupled with a lack of effective elimination of errors, such as atmospheric errors, the accuracy of the obtained absolute deformation is lacking [17]. In general, the above three techniques have provided a large amount of deformation data for the investigation of the formation mechanism of ground fissures. However, as surface monitoring technologies, these three techniques are hindered by topography and land cover, and this may produce unreliable monitoring results [18]. Therefore, it is of great importance to develop a more effective technique to obtain the accurate deformation data inside the monitored ground fissure, which can be used to make activity analysis.

Extensometers, strainmeters, and tiltmeters are the commonly used instruments to perform real-time deformation monitoring of ground fissures. Extensometers can be used to obtain more precise and accurate data of land elevation changes and vertical deformation of ground fissures with a resolution of 0.01–0.1 mm [19,20]. Strainmeters and tiltmeters can be used to measure the horizontal changes in the local tilt and stress-strain of ground fissures, which allow obtaining high precision measurements [21]. The shape acceleration array (SAA) is a sensor based on a micro-electro-mechanical system (MEMS) that is mounted at a certain depth of less than 100 m of a monitored ground fissure. Compared with extensometers, strainmeters, and tiltmeters, SAA sensors can be used to obtain 3D displacements accurately and continuously with a high temporal resolution (data acquisition frequency up to 1 Hz) [22,23], and the fine movements prior to failure can be captured reliably using SAA sensors [24]. These sensors can reliably meet the deformation testing requirements of static geotechnical engineering, such as sliding slope, tunnel, and embankment settlement [25,26]. Moreover, previous studies have shown that landslides and ground fissures have great similarities, both of which have the characteristics of slow change, concealment, and random suddenness [23]. They are also affected by various environmental factors and stresses, and the displacement change follows a certain periodic law [23,27]. David Huntley et al. [28] used SAA sensors to measure the subsurface inclinometry of the Ripley Slide in the Thompson River Valley south of Ashcroft, British Columbia. Macciotta et al. [26] designed an early warning system based on SAA and GPS measurements that focused on detecting changes in landslide annual displacement cycles and potential accelerations, as well as the effects of slope deformation on railway alignment using an application to monitor the Ripley Landslide at two important railroad crossings. Journault et al. [29] presented a comparison of the InSAR measurements using SAA installed at a moving landslide to address the extent and magnitude of the slope movements observed. The results showed that similar displacement values were obtained by these two measurements. Chang Ping-Yu et al. [30] used SAA sensors to monitor the long-term creeping of the Lichi Mélange along the hill slope. They designed the sensors to probe into the spatial structures of the frontal deformation zone.

The objective of this study is to use the instantaneous total energy method to perform an activity analysis of a monitored ground fissure based on the accurate time-series displacement acquired by standard SAA sensors, and further to provide safety information regarding the monitored ground fissure. The instantaneous total energy is proposed to analyze the activity of the monitored ground fissure by integrating the extreme-point symmetric mode decomposition (ESMD) method and kinetic energy, which is based on the time-series displacement acquired by SAA sensors. The Xiwang Road ground fissure located in Beijing, China, is selected as a test site. SAA sensors are embedded below the surface crossing the monitored ground fissure to obtain accurate time-series displacement data.

## 2. Study Site

**Figure 1:**
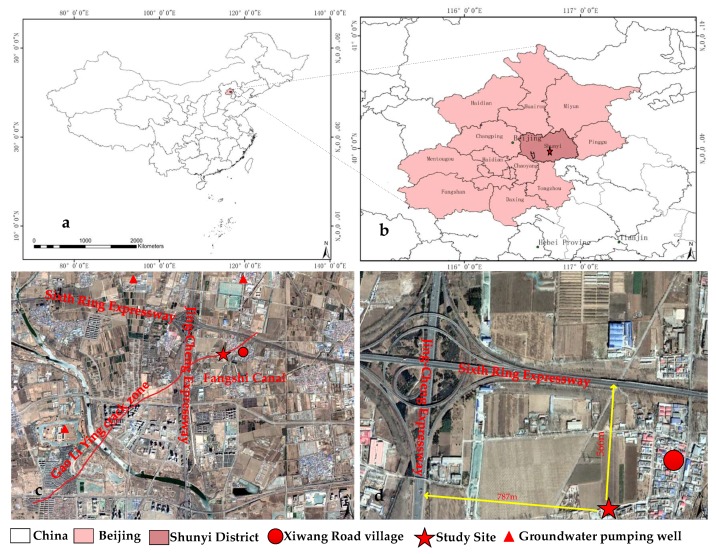
Location of the study site, (**a**) the study site in China, (**b**) the study site in Shunyi District of Beijing, (**c**) two expressways, a canal, three pumping wells and Gao Li Ying crack zone around the study site, (**d**) distances between two expressways and the study site.

The Xiwang Road ground fissure is primarily located in Xiwang Road Village along the Huang Zhuang-Gao Li Ying fault in Shunyi District of Beijing, China, as shown in Figure 1, which was first discovered in the early 1990s. It is adjacent to the Jing-Cheng Expressway in the west with a distance of 787 m, the Sixth Ring Expressway in the north with a distance of 566 m, and the Fangshi Canal approximately 20 m in width in the south. In addition, an intersection area of the ground fissure between the Jing-Cheng Expressway and the Xiwang Road consists primarily of woodlands and farmlands, and there are nurseries and villages on the east side of the expressway, as shown in Figure 1. Moreover, there are three groundwater pumping wells in the west and north of the monitored ground fissure, as shown in Figure 1.

The Xiwang Road ground fissure is controlled by the Huang Zhuang-Gao Li Ying fault, and its deformation is the result of fault creep deformation and the differential settlement caused by groundwater recession [31]. The study site has strong quaternary activities that control the quaternary subsidence centers in the Huairou and Shunyi districts. Shallow seismic exploration revealed that the Huang Zhuang-Gao Li Ying fault played a dominant role in the generation and development of the monitored ground fissure, and the Xiwang Road ground fissure was the extension of fault activities in the front of the ground surface [32]. In addition, the quaternary aquifer is composed of a phreatic layer and a confined aquifer. Confined aquifer is the main aquifer of the study site, which is mainly recharged by surface water and upper phreatic aquifer. However, with the massive exploitation of groundwater in recent years, the confined aquifer has been declining year by year, with a cumulative decline in height of 9 m. Moreover, with the changes of precipitation and exploitation, the fluctuation range of the water level in confined aquifer varies greatly, which is an inducing factor for the development of the monitored ground fissure. At present, the Xiwang Road ground fissure has a horizontal offset of several to hundreds of millimeters, in which the maximum horizontal offset is 200 mm, and it has a vertical offset of several to dozens of centimeters, in which the maximum vertical offset is 53 cm. The maximum vertical displacement rate of the monitored ground fissure is 18 mm/year in the west and north of the monitored ground fissure [32]. Moreover, after a heavy rain in the summer of 2001, five caves were generated in the area of the Xiwang Road ground fissure. These had interrupted the normal operation of the surrounding highways and caused significant damage to the walls and floors of many local houses. The Xiwang Road ground fissure has seriously endangered the safety of people and property in the area.

## 3. SAA Sensor Layout

**Figure 2:**
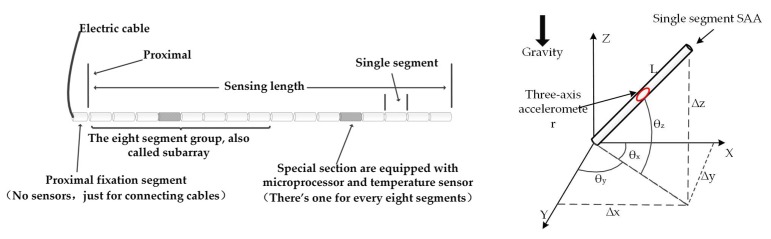
Structure and principle of a shape acceleration array (SAA) sensor.

In this study, the wireless SAA sensors, developed by a collaboration of a team of researchers at the Rensselaer Polytechnic Institute (RPI) and Measurand, Inc., were used to obtain the time-series displacement of the monitored ground fissure. The concept of the sensors is based on MEMS accelerometer measurements of angles relative to gravity, which also provide signals proportional to vibrations during construction activities. The sensors were contained in 30 cm-long rigid segments that were connected using composite joints that prevent torsion but allow flexibility of two degrees of freedom [25]. As shown in Figure 2, each SAA basic unit consists of one cable, seven joints, and eight sensor segments with a standard length of 500 mm. Each sensor segment is equipped with an accelerometer and a microprocessor, and the fourth segment has an added temperature sensor. Each SAA sensor segment contains a set of three axis accelerometers. By detecting the gravity field, the bending angles, θ, between the axes of each segment are obtained. Using the obtained bending angle, θ, and the known segment length, L, the deformation of each segment is obtained using Δd=θ×L.

**Figure 3:**
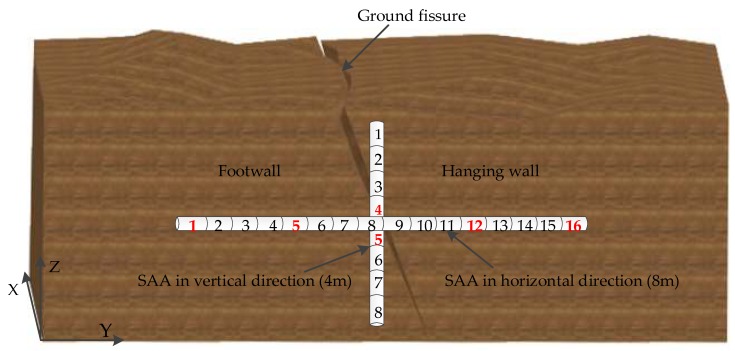
Layout of the SAA sensors at the Xiwang Road ground fissure.

**Figure 4:**
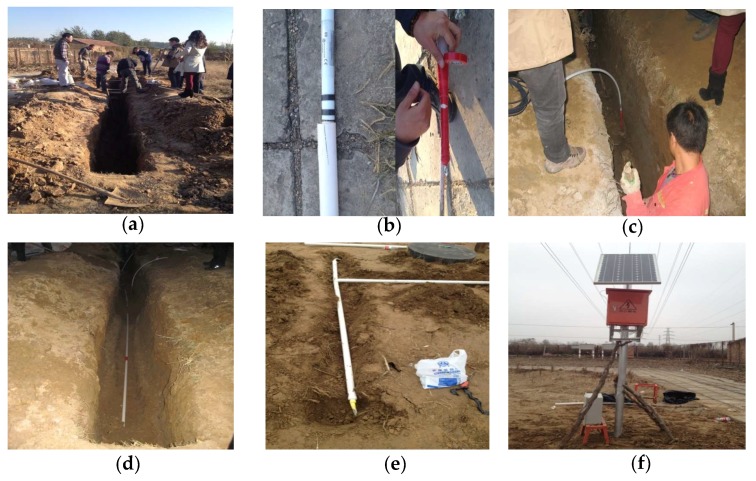
Charts of the SAA installation process. (**a**) Excavated foundation pit; (**b**) Schematic diagram of fixed protection for SAA sensors; (**c**) Vertically embedded SAA sensor in the ground fissure; (**d**) Horizontally embedded SAA sensor in the ground fissure; (**e**) Buried polyvinyl chloride (PVC) pipes for data connection protection; (**f**) Solar energy storage devices and data acquisition devices.

As shown in Figure 3, like a fault, a ground fissure can also be divided into a hanging wall and a footwall. SAA sensors are normally embedded below the surface to obtain real-time displacements and depth changes. The sensors are installed inside polyvinyl chloride (PVC) pipes that have inner and outer diameters of 25 and 32 mm, respectively [24]. In this study, to fully monitor the hanging wall and footwall of the Xiwang Road ground fissure and analyze its activity, two SAA sensors were embedded in the Xiwang Road ground fissure with an acquisition frequency of once an hour that included a 4 m-long SAA with eight nodes in the vertical direction and an 8 m-long SAA with 16 nodes in the horizontal direction, as shown in Figure 3. The *X*-axis is parallel to the direction of the monitored ground fissure, and this indicates the horizontal twist direction of the ground fissure. The *Y*-axis is perpendicular to the ground fissure, and this indicates the direction of horizontal cracking of the ground fissure. The *Z*-axis is perpendicular to the ground surface and indicates the direction of ground fissure subsidence. The detailed process of SAA sensor installation is shown in Figure 4 and can be summarized in the following steps:

(1) As shown in Figure 4a, centered on the ground fissure, a foundation pit with a length of 8.5 m and a width of 0.5 m was excavated along the direction of the vertical ground fissure with a depth of 0.5 m under backfill. A vertical hole with a diameter of 0.1 m and a depth of 4 m was drilled in the middle of the foundation pit near the hanging wall of the ground fissure.

(2) SAA sensors were loaded into PVC pipes of suitable sizes, and the two ends were fixed and protected using waterproof adhesive tape, as shown in Figure 4b.

(3) A 4 m long SAA sensor was vertically embedded in the original soil layer across the upper and lower plates to monitor the relative displacement of the ground fissure, as shown in Figure 4c. An 8-m-long SAA sensor was horizontally embedded in the original soil layer to monitor the relative displacement of the ground fissure, as shown in Figure 4d.

(4) The foundation pit was backfilled using fine soil, and the data connection line was protected and buried using PVC pipes, as shown in Figure 4e. Finally, solar energy storage devices and data acquisition devices were installed to obtain the monitored time-series displacement, as shown in Figure 4f.

## 4. Methodology

**Figure 5:**
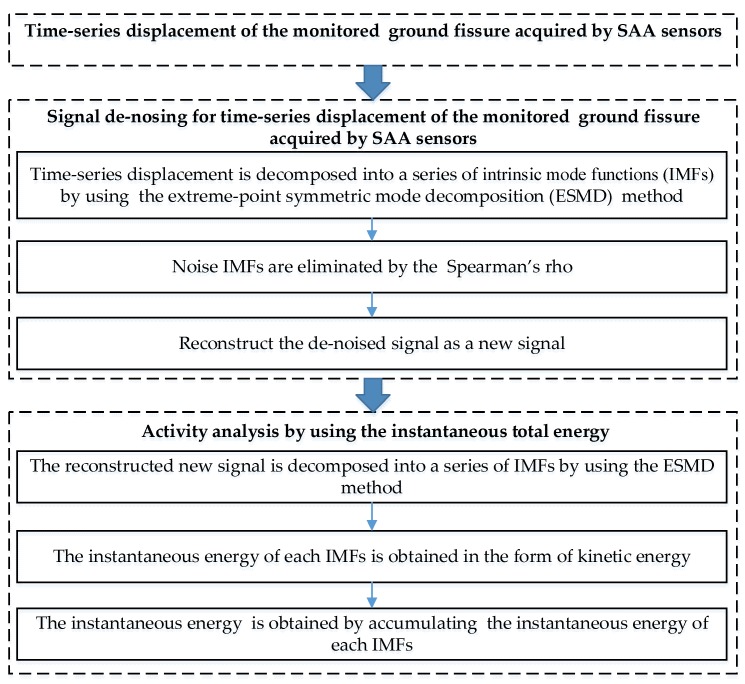
Workflow of the activity analysis using the instantaneous total energy.

Figure 5 shows the entire technical framework for the activity analysis of the monitored ground fissure. This consisted of the following two steps: Signal denoising of the time-series displacement acquired using SAA sensors of the monitored ground fissure, and an activity analysis that used the instantaneous total energy calculated by the denoised time-series displacement.

### 4.1. Signal Denoising Using the ESMD Method and Spearman’s Rho

In this study, during the period of ground fissure monitoring using the SAA sensors, noise is inevitably generated by various factors such as heavy traffic, ground motion, and other factors. These events will cause some loss in precision of the obtained time-series displacements. Generally, a complicated response signal can be regarded as being composed of multiple simple sub-signals, which include useful signals and noise [33,34]. The empirical mode decomposition (EMD) denoising method has been widely used for signal denoising [35,36]. However, the EMD method has a mode mixing problem of the decomposed IMFs that leads to both useful components and noise being contained in any decomposed IMF [36]. The extreme-point symmetric mode decomposition (ESMD) method is a new alternative time-frequency analysis method that has the ability to decompose any complicated original signal into a collection of band-limited quasi-stationary IMFs by determining an optimal adaptive global mean (AGM) curve [37]. Compared with the EMD method, the ESMD method has displayed better performance in decomposing a signal into a series of physically meaningful representations [34]. Additionally, correlation analysis is an important application in signal noise processing [38]. Spearman’s rho is a nonparametric coefficient correlation analysis method that only depends on ranks when calculating data correlations, hence it has improved signal noise processing robustness [39]. Therefore, with the aim to improve the reliability of the activity analysis using the instantaneous total energy, this study integrated the ESMD method and Spearman’s rho to perform signal denoising of the original time-series displacement data obtained using the SAA sensors.

For a given time-series displacement signal, s(t), the primary steps of signal denoising using the ESMD method and Spearman’s rho are as follows.

(1) Find all the local maxima and minima points of the signal, s(t), and enumerate the midpoints of the adjacent extreme points as $M_{i}\;(i\;=\;1,2,\cdots ,n-1)$. Then, for the odd and even midpoints in $M_{i}$, construct two curves, $L_{1}$ and $L_{2}$, using cubic B-spline interpolation, and further calculate their mean curve, $\bar{L}=\left(L_{1}+L_{2}\right)/2$.

(2) Denote $\bar{s}$ as the average value of the original signal, s(t). Determine $\sigma_{0}=\sqrt{\frac{1}{n}{\sum_{t=1}^{n}\left(s(t)-\bar{s}\right)}^{2}}$ as the variance of the original signal, s(t), relative to the total mean, $\bar{s}=\left(\sum_{t=1}^{n}s(t)\right)/n$, and set the permitted error to $\varepsilon =0.001\sigma_{0}$ to guarantee the quality of the IMF decomposition.

(3) Determine an optimal sifting times term, $K_{o}$, to avoid an endless loop of IMF decomposition with the unique permitted error, ε. The procedure is as follows: (a) Preset a maximum sifting times value, $K_{\max}$, and repeat steps (1) and (2) on $s(t)-\bar{L}$ until $\left|\bar{L}\right|\leq \varepsilon$ to obtain IMF1; (b) repeat the above steps on s(t)−IMF1 to obtain IMF2, IMF3, and so on until the AGM curve, R=r(t), has less than a certain number of extreme points. Then calculate the variance, σ, of s(t) relative to the AGM curve, R, using $\sigma =\sqrt{\frac{1}{n}{\sum_{t=1}^{n}\left(s(t)-r(t)\right)}^{2}}$; and (c) change the sifting times value, K, on a finite integer interval $\left[1,K_{\max}\right]$. Then repeat steps (a) and (b) to obtain the optimal sifting time term, $K_{o}$, according to the minimum, $\sigma_{\min}$, on $\left[1,K_{\max}\right]$.

(4) Perform the optimal signal decomposition for the time-series displacement signal, s(t), to obtain a series of IMFs with an optimal AGM curve using the optimal sifting times value, $K_{o}$ and the permitted error, σ.

(5) Perform signal denoising by eliminating the noise dominant IMFs from the decomposed IMFs using Spearman’s rho. Spearman’s rho can be used to express the correlation degree between two signals [39]. The Spearman’s rho is between [−1, +1] between the decomposed IMFs and the original signal. The smaller Spearman’s rho, the more noise information of each decomposed IMF. Therefore, noise dominant IMFs have less similar features than the original signal. Assume that the original signal is decomposed into m IMFs, and denote $IMF_{j}\left(t\right)\left(1\leq j\leq m\right)$ as the jth decomposed IMF from the original signal. The principle for determining the removal of noise dominant IMFs is to find the $IMF_{j}\left(t\right)$, of which Spearman’s rho turns in the opposite direction. This is then the cut-off point of the similarity between the decomposed IMFs and the original signal, which displays a gradual decrease to gradual increase [40,41]. At last, the denoised signal is reconstructed by deleting $IMF_{1}\left(t\right)$ to $IMF_{j}\left(t\right)$. The Spearman’s rho between each decomposed IMF and the original signal can be calculated using Equation (1):

(1) $$\rho \left(s(t),IMF_{j}\left(t\right)\right)=1-\frac{6{\sum_{t=1}^{n}\left(s(t)-IMF_{j}\left(t\right)\right)}^{2}}{n\left(n-1\right)},$$

where, $\rho \left(s(t),IMF_{j}\left(t\right)\right)$ is the Spearman’s rho between the decomposed $IMF_{j}\left(t\right)$ and the original signal s(t).

### 4.2. Instantaneous Total Energy of the Activity Analysis

The Fourier frequency-spectrum and Hilbert time-frequency-spectrum are the most used spectrum methods for total energy analysis, and these project the total energy onto a series of frequencies [37,42,43]. However, the frequency and instantaneous energy of each decomposed IMF shift at any time, and the resulting instantaneous total energy itself changes with time. With this understanding, the spectrum methods of the Fourier frequency-spectrum and the Hilbert time-frequency-spectrum are unreasonable. Generally, a ground fissure should be affected by multiple external forces. If there is a dominant external force, or there are multiple external forces acting on a ground fissure in the same direction, there will be a large time-series displacement. However, if there are two dominant approximately equal external forces acting on a ground fissure in the opposite direction, these will produce a very small time-series displacement, but it is a strong activity for the monitored ground fissure. With this understanding, it is unreasonable to analyze the activity of a ground fissure by directly using time-series displacement. Therefore, the time-variation of the instantaneous total energy is proposed to analyze the activity of a monitored ground fissure. In this study, instantaneous total energy can be understood as an entire activity intensity of the ambient excitation of each IMF decomposed from the time-series displacement of a monitored ground fissure. Denote $IMF_{j}\left(t\right)\left(1\leq j\leq m\right)$ as the jth decomposed IMF from the original signal, s(t), using the ESMD method and Spearman’s rho, and use $A_{j}\left(t\right)\left(1\leq j\leq m\right)$ as the amplitude curve of real oscillations corresponding to $IMF_{j}\left(t\right)$. In addition, denote G as mass of a plate of a monitored ground fissure, and Δt as time interval of adjacent data acquisition. The instantaneous energy of $IMF_{j}\left(t\right)$ of a monitored ground fissure can be defined in the form of kinetic energy, as in Equation (2). Moreover, in order to simplify the expression of instantaneous energy, here, assume that G is 1 and Δt is 1. Therefore, the instantaneous energy of $IMF_{j}\left(t\right)$ of a monitored ground fissure can be defined using Equation (3). The instantaneous total energy of the original signal, s(t), can be described using Equation (4).

(2) $$E_{j}(t)=\frac{1}{2}{\sum_{t=1}^{n}G\left(\frac{A_{j}\left(t\right)}{\Delta t}\right)}^{2}$$

(3) $$E_{j}(t)=\frac{1}{2}\sum_{t=1}^{n}A_{j}{}^{2}\left(t\right)$$

(4) $$E(t)=\sum_{j=1}^{m}E_{j}\left(t\right)$$

## 5. Results and Discussion

### 5.1. Results of Variations in the Ground Fissure

**Figure 6:**
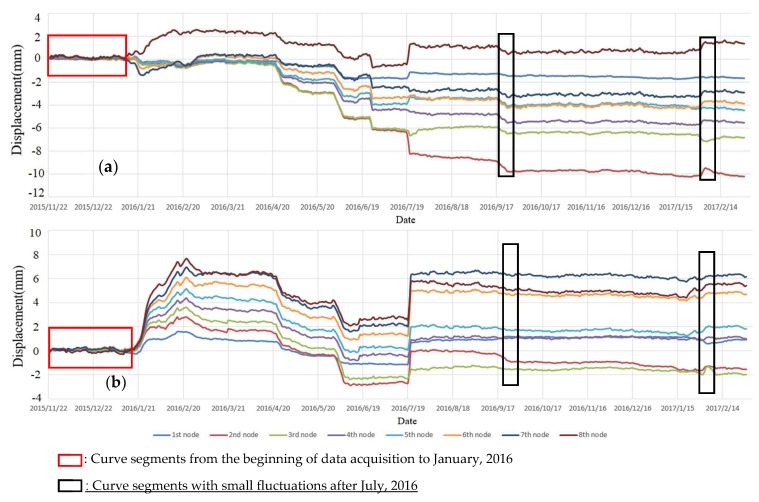
Curves of cumulative horizontal displacement in the X and Y directions of the 4 m-long SAA. (**a**) Curves of cumulative horizontal displacement in the X direction of the 4 m-long SAA; (**b**) Curves of cumulative horizontal displacement in the Y direction of the 4 m-long SAA.

Figure 6 shows the cumulative displacement curves of eight nodes (see in Figure 3) of the 4 m-long SAA in the *X* and *Y* directions using the daily average displacement. An inspection of this figure highlights the following: (1) From the beginning of data acquisition to January 2016 (curve segments in the red box shown in Figure 6), there were no obvious changes in the displacement curves of each node in the *X* and *Y* directions of the 4 m-long SAA. Moreover, although there were two small fluctuations (curve segments in the four black boxes shown in Figure 6) in the displacement curves in the *X* and *Y* directions of the eight nodes of the 4 m-long SAA after July 2016, the displacement curves of the eight nodes changed smoothly. Therefore, the monitored ground fissure was considered stable during these periods of time. (2) From January to July 2016, the displacement curve of each node gradually became larger. These results show that the fissure may have been in an active phase of deformation during this period. Moreover, between April and July, sharp changes of displacement occurred, with a maximum displacement of 8.2 mm in the *X* direction for the eight nodes. Between January and July 2016, sharp changes in the displacement occurred, with a maximum displacement of 7.9 mm in the *Y* direction for the eight nodes. As shown in Figure 3, the *X* direction represents the horizontal torsion direction, and the *Y* direction represents the horizontal tension direction. Therefore, the monitored ground fissure first stretched significantly in the *Y* direction, which caused torsion on the monitored ground fissure in the *X* direction. These results show that tension may be the primary factor in the deformation of monitored ground fissures.

**Figure 7:**
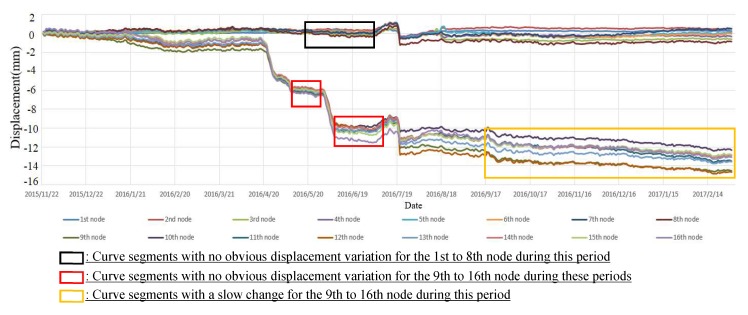
The cumulative horizontal displacement curve of the SAA in the Z direction of the 8 m-long SAA.

Figure 7 shows the cumulative displacement curves in the Z direction using the daily average displacement of 16 nodes (see in Figure 3) of the 8 m-long SAA. An inspection of this figure highlights the following points: (1) For the displacement curves of the 1st to 8th node placed in the footwall, the maximum displacement was less than 0.6 mm, which can be considered stable. However, for the displacement curves of the 9th to 16th node placed in the hanging wall, there was a large vertical displacement with a maximum displacement of more than 12 mm from April to July 2016. In addition, the displacement of each node also tended to increase gradually from July 2016 to the end of monitoring. These results show that the hanging wall of the monitored ground fissure was in an active deformation phase during this period of time. (2) As shown in Figure 6 and Figure 7, the displacement curve of each node of the 4 m-long and 8 m-long SAAs had obvious simultaneous mutations from 19–21 July 2016. According to historical meteorological data, a rainstorm occurred on that day may have caused a plate dislocation.

### 5.2. Signal Denoising

**Figure 8:**
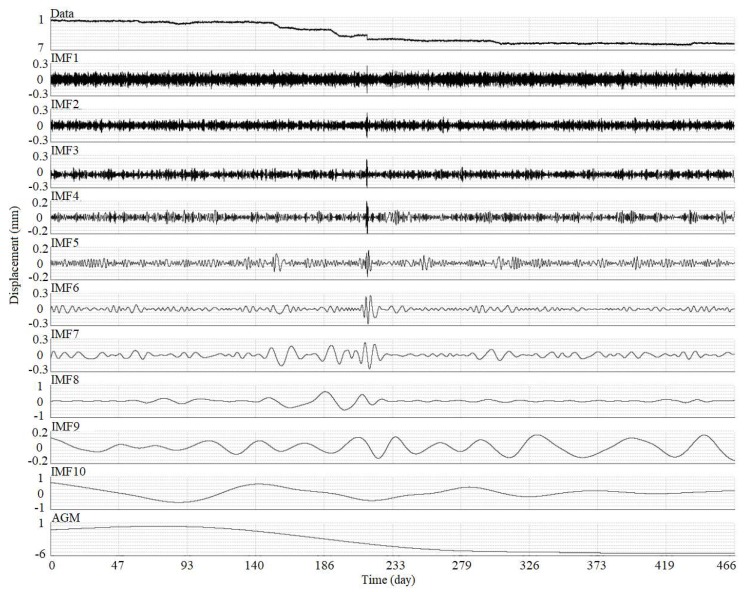
Decomposed intrinsic mode functions (IMFs) using the ESMD method for the time-series displacement in the *X* direction of the 4th node of the 4 m-long SAA.

**Figure 9:**
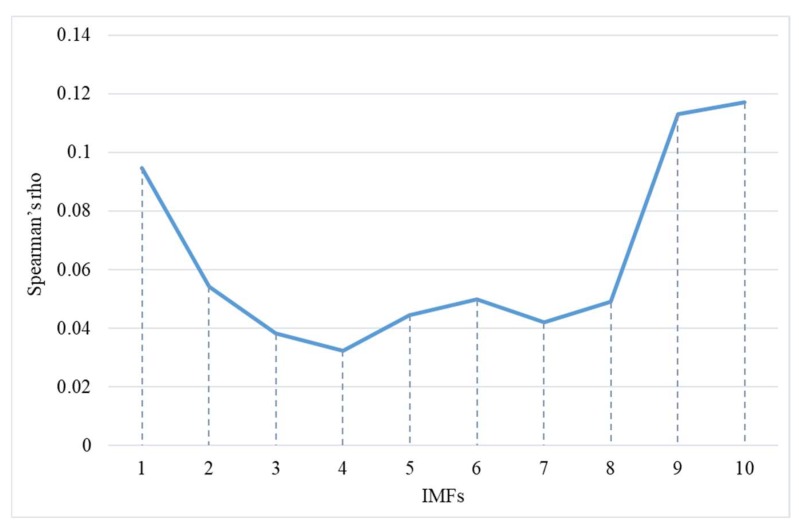
Spearman’s rho of each decomposed IMF.

**Figure 10:**
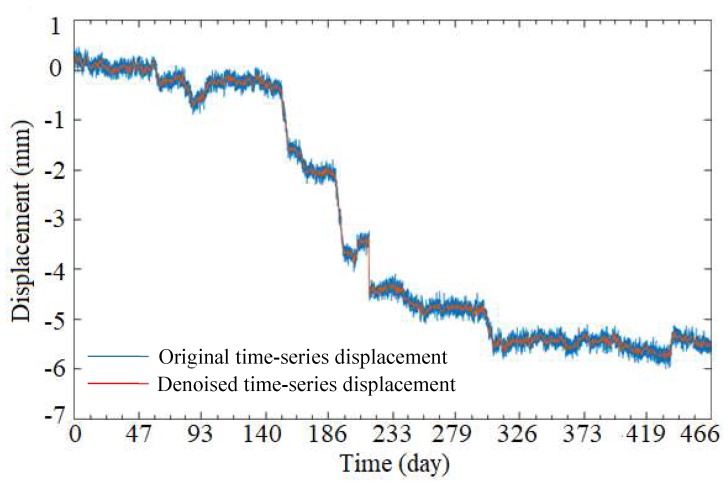
Comparison between the original signal and the denoised signal.

To improve the accuracy of the activity analysis using instantaneous total energy, the original time-series displacement was denoised using the ESMD method and Spearman’s rho [34]. In this study, the time-series displacement in the *X* direction of the 4th node (see in Figure 3) of the 4 m-long SAA was selected for the experimental data, as shown in Figure 8. Using the ESMD method, the original time-series displacement was decomposed into 10 IMFs with the corresponding meaningful inherent natural frequency from high to low frequency, as shown in Figure 8. The Spearman’s rhos between each decomposed IMF and the original time-series displacement are shown in Figure 9. The minimal value of Spearman’s rho was 0.324 between the IMF4 and the original time-series displacement. Therefore, the first four IMF components, IMF1 to IMF4, were regarded as the noise dominant IMFs, which may be caused by instrument errors, ground motion, heavy traffic, and other factors. The remaining IMF components were regarded as useful sub-signals to be reconstructed as a new denoised signal, as shown in Figure 10. However, in this study, it is difficult to determine the specific meaning of each decomposed IMF. It just has an ability to distinguish the noise dominant IMFs and useful IMFs from all of the decomposed IMFs. For the denoised signal, the signal-to-noise ratio (SNR) was 28.5 dB, which was an improvement of 11.3% compared with an SNR of 25.6 dB for the original signal. In addition, the root-mean-square error (RMSE) was 0.088 mm, which has an improvement of 9.3% compared with an RMSE of 0.097 mm for the original signal.

### 5.3. Analysis of Ground Fissure Activity

**Figure 11:**
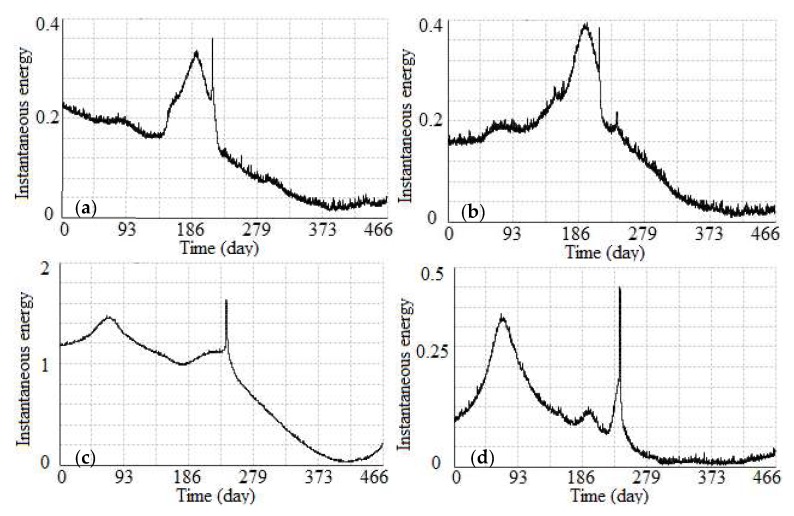
Instantaneous total energy of the 4th and 5th nodes in the *X* and *Y* directions of the 4 m-long SAA. (**a**) *X* direction of the 4th node; (**b**) *X* direction of the 5th node; (**c**) *Y* direction of the 4th node; (**d**) *Y* direction of the 5th node.

For the cumulative displacement curves in the *X* and *Y* directions of eight nodes of the 4 m-long SAA, the 4th and 5th nodes (see in Figure 3) were selected to conduct an activity analysis using the instantaneous total energy, as shown in Figure 11. An inspection of the instantaneous total energy in this figure clearly highlights the following: (1) For the instantaneous total energy of the 4th and 5th nodes in the *X* direction, both displayed a similar slow growing process from approximately the 120th day (April 2016) to approximately the 190th day (June 2016). These results indicate that both the hanging wall and footwall were in an active stage in the *X* direction during this period of time. (2) For the instantaneous total energy of the 4th and 5th nodes in the *Y* direction, both of them had a similar slow growing process from the beginning to approximately the 70th day (January 2016). These results indicate that both the hanging wall and footwall were in an active stage in the Y direction during this period of time. However, the value of the instantaneous total energy of the 4th node was greater than that of the 5th node, which indicates that hanging wall was more active in the *Y* direction than the footwall during this period of time. (3) For the instantaneous total energy of the 4th and 5th nodes in the *X* and *Y* directions, there was a transient mutation from the 241th day to 243th day (19–21 July 2016). According to historical meteorological data, a heavy rainstorm occurred that day and was the cause of severe plate movement. Furthermore, after 21 July 2016, a rapid decline process occurred in the instantaneous total energy of the 4th and 5th nodes in the *X* and *Y* directions. Hence, both the hanging wall and footwall gradually transitioned to a stable stage.

**Figure 12:**
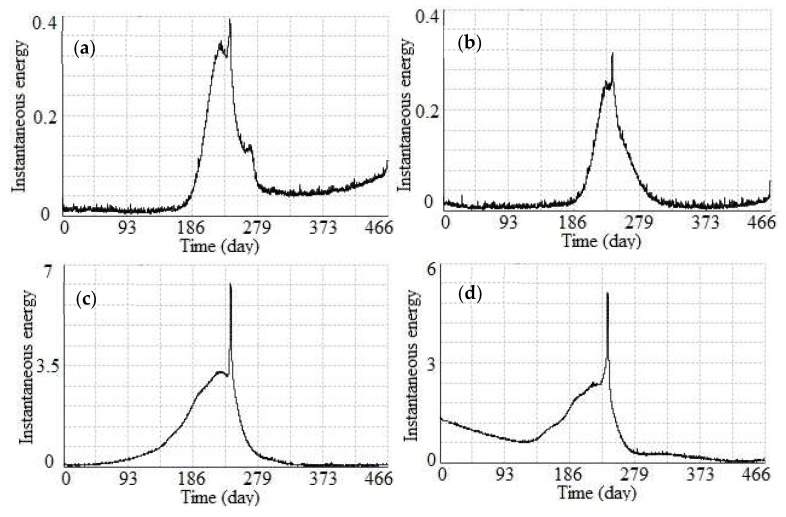
Instantaneous total energy in the *Z* direction of the 8 m-long SAA. (**a**) 1st node, (**b**) 5th node, (**c**) 12th node, and (**d**) 16th node.

The 1st, 5th, 12th, and 16th nodes (see in Figure 3) were selected to conduct an activity analysis using the instantaneous total energy, as shown in Figure 12. An inspection of the instantaneous total energy in this figure clearly highlights the following: (1) For the instantaneous total energy in the *Z* direction of the 1st and 5th nodes, both displayed a similar slow growing process from approximately the 180th day (May 2016) to approximately the 220th day (July 2016), which indicates that the footwall was in an active stage in the *Z* direction during this period of time. However, as the black box shown in Figure 7 indicates, there was no obvious change in the displacement curves in the *Z* direction of the 1st and 5th nodes during this period of time, which indicates that the instantaneous total energy more accurately reflected the activity of the monitored ground fissure compared with the time-series displacement. (2) For the instantaneous total energy in the *Z* direction of the 12th and 16th nodes, both had a similar slow growing process from approximately the 150th day (April 2016) to approximately the 220th day (July 2016). These results indicate that the hanging wall was also in an active stage in the Z direction during this period of time. Furthermore, the value of the instantaneous total energy of the 12th and 16th nodes was larger than 1st and 5th nodes, which indicates that the active intensity of the hanging wall was greater than that of the footwall. In addition, as the two red boxes shown in Figure 7, although the displacement curves of the 12th and 16th nodes were flat in the Z direction during this period of time, there was an increasing trend in the corresponding instantaneous total energy. As the yellow box shown in Figure 7, there is a slow change in the displacement curves of the 12th and 16th nodes in the *Z* direction during this period of time, but the corresponding instantaneous total energy is smaller, which indicates the footwall was in a stable stage. These results also show that the instantaneous total energy more accurately reflected the activity of the monitored ground fissure compared with the time-series displacement. (3) For the instantaneous total energy in the *Z* direction of these 4 nodes, there was also a transient mutation from the 241th day to 243th day (19–21 July 2016), which was also caused by a heavy rainstorm day. In addition, after 21 June 2016, both the hanging wall and the footwall gradually attained a stable stage.

By the ESMD method, each decomposed sub-signal has a different own inherent natural frequency [44]. That is to say, each simple signal is produced by a different external force. For the monitored ground fissure, the obtained time-series displacement may have been caused by some external forces, such as a geological tectonic movement, a temperature difference, surrounding construction, groundwater mining, and precipitation, which would have increased the activity in the monitored ground fissure [31]. Therefore, in this study, the ESMD method was used to decompose the time-series displacement into a series of IMFs. In addition, the instantaneous total energy along time was obtained by accumulating the instantaneous energy of each IMF, which is more reasonable than the spectrum methods [43]. As the red boxes shown in Figure 7, there is no obvious displacement variation for each node in the *Z* direction during these periods, but there is an obvious increasing trend for the corresponding instantaneous total energy. The reason is that there are some external forces acted on the monitored ground fissure, but the directions of the external forces were opposite, which cannot bring obvious changes in displacement. Therefore, the instantaneous total energy is a more reliable index compared to the time-series displacement to reflect the activity of a monitored ground fissure.

As shown in Figure 11 and Figure 12, between April and July 2016, the instantaneous total energy of each node in the *X*, *Y* and *Z* directions tends to increase gradually, especially for the nodes in the *Z* direction of the 8 m-long SAA. As we know, the monitored ground fissure is in a semi-humid and semi-arid continental monsoon climate in a warm temperate zone, of which rainy season is from April to July each year. Therefore, due to the massive exploitation of groundwater, a greater variability of water level in confined aquifer will be caused by groundwater recharging in rainy season, which can cause great changes in instantaneous total energy. Especially for the period from 19–21 July 2016, a transient mutation of instantaneous total energy was caused by a heavy rainstorm day, which is a process of quantitative change to qualitative change to cause severe plate movement caused by groundwater recharging. This indicates that groundwater recharging is an inducing factor for the motions of the monitored ground fissure. In addition, although there is a smaller fluctuation in the instantaneous total energy of each node in the *X*, *Y* and *Z* directions in dry season, it is a small value of instantaneous total energy, which indicates that the monitored ground fissure is in a relative stable state in dry season. Moreover, the temperature differences between day and night are large from April to June each year, which is basically consistent with the time period of the active ground fissure. Therefore, ground fissures will expand and contract under the influence of temperature differences, and the instantaneous total energy will significantly change when the temperature difference between day and night is large.

## 6. Conclusions

To accurately perform an activity analysis of ground fissures, this study proposed a novel method that used the instantaneous total energy based on time-series displacement data. The time-series displacement data were obtained using SAA sensors. The ESMD method and Spearman’s rho were integrated to perform signal denoising to improve the accuracy of the obtained time-series displacement data. The instantaneous total energy was proposed to analyze the activity of the monitored ground fissure. More specifically, the results of this study reported the following conclusions:

(1) Aiming to obtain the accurate time-series displacement inside the monitored ground fissure, with the advantages of 3D measurement and a high temporal resolution, this study used SAA sensors to be embedded in a monitored ground fissure to acquire the accurate time-series displacement in the three directions, which included a 4 m-long SAA in the vertical direction and an 8 m-long SAA in the horizontal direction. The results showed the SAA sensors are a reliable instrument to fully monitor the hanging wall and footwall of the monitored ground fissure.

(2) Aiming to reduce the influence of noise for the obtained time-series displacement using SAA sensors, this study integrated the ESMD method and Spearman’s rho to perform signal denoising to decrease the effects of noise in the time-series displacement data. The ESMD method was used to decompose the time-series displacement data into a series of IMFs with different frequency scales. The noise dominant IMFs can be determined by Spearman’s rho, and the remaining IMF components can be reconstructed as a new denoised signal. The experimental results showed that the proposed method reduced the impact of noise effectively with an improvement of more than 10% in the SNR and RMSE compared with the original time-series displacement data. However, it just has an ability to distinguish the noise dominant IMFs and useful IMFs from all of the decomposed IMFs. It is difficult to determine the specific meaning of each decomposed IMF.

(3) Aiming to accurately analyze the activity of a monitored ground fissure, this study proposed a novel idea of the instantaneous total energy to perform an activity analysis of a monitored ground fissure. The ESMD method was first used to decompose the denoised time-series displacement into a series of IMFs, which represent different external forces, and then the instantaneous total energy with time was obtained by accumulating the instantaneous kinetic energy of each IMF. If multiple external forces act on a ground fissure in the approximately same directions, both of the time-series displacement and instantaneous total energy can accurately reflect the activity of the monitored ground fissure. Otherwise, the instantaneous total energy is more reasonable to reflect the activity compared to the time-series displacement. The experimental results showed that the instantaneous total energy was a more reliable index to reflect the activity of a monitored ground fissure compared to the time-series displacement.
